# Spillover of pH1N1 to swine in Cameroon: an investigation of risk factors

**DOI:** 10.1186/1746-6148-10-55

**Published:** 2014-03-04

**Authors:** Brenda Larison, Kevin Y Njabo, Anthony Chasar, Trevon Fuller, Ryan J Harrigan, Thomas B Smith

**Affiliations:** 1 Center for Tropical Research, Institute of the Environment and Sustainability, University of California, 619 Charles E. Young Drive East, Los Angeles, California 90095, USA; 2 Department of Ecology and Evolutionary Biology, University of California, 610 Charles E. Young Drive South, Los Angeles, California 90095, USA

## Abstract

**Background:**

The 2009 pH1N1 influenza pandemic resulted in at least 18,500 deaths worldwide. While pH1N1 is now considered to be in a post-pandemic stage in humans it has nevertheless spilled back into swine in at least 20 countries. Understanding the factors that increase the risk of spillover events between swine and humans is essential to predicting and preventing future outbreaks. We assessed risk factors that may have led to spillover of pH1N1 from humans to swine in Cameroon, Central Africa. We sampled swine, domestic poultry and wild birds for influenza A virus at twelve sites in Cameroon from December 2009 while the pandemic was ongoing, to August 2012. At the same time we conducted point-count surveys to assess the abundance of domestic livestock and wild birds and assess interspecific contact rates. Random forest models were used to assess which variables were the best predictors of influenza in swine.

**Results:**

We found swine with either active pH1N1 infections or positive for influenza A at four of our 12 sites. Only one swine tested positive by competitive ELISA in 2011-2012. To date we have found pH1N1 only in the North and Extreme North regions of Cameroon (regions in Cameroon are administrative units similar to provinces), though half of our sites are in the Central and Western regions. Swine husbandry practices differ between the North and Extreme North regions where it is common practice in to let swine roam freely, and the Central and Western regions where swine are typically confined to pens. Random forest analyses revealed that the three best predictors of the presence of pH1N1 in swine were contact rates between free-ranging swine and domestic ducks, contact rates between free-ranging swine and wild Columbiformes, and contact rates between humans and ducks. Sites in which swine were allowed to range freely had closer contact with other species than did sites in which swine were kept penned.

**Conclusions:**

Results suggest that the practice of allowing swine to roam freely is a significant risk factor for spillover of influenza from humans into swine populations.

## Background

The H1N1 2009 influenza pandemic resulted in hundreds of thousands of human cases worldwide [1]. To date the pandemic has resulted in 18,500 lab-confirmed deaths [1,2], though estimating the total mortality of the pandemic may take years. While now in a post-pandemic period [3], an understanding of the transmission and spillover dynamics of H1N1 remains essential for the prediction and prevention of future pandemics.

Pandemic influenza A (pH1N1) is a swine-origin influenza virus (S-OIV) that may have been circulating among humans for several months before becoming pandemic [4]. Swine were not implicated in the spread of the virus during the pandemic [5], but infected swine have subsequently been found in 20 countries [6-9] including Cameroon [10]. The public health importance of influenza infections in swine arises from the fact that swine are susceptible to co-infections with multiple lineages of the influenza virus, which can generate novel strains via reassortment [8,11]. Reassortant viruses containing genes from pH1N1 and other influenza subtypes have already been isolated from swine in China, the United States, and the United Kingdom [7,12,13]. As a consequence there is concern that the next pandemic strain could arise in swine, although the spread of reassortant virus among humans would require further adaptation in order to replicate efficiently in humans and spread between them [14,15].

In August 2009, pH1N1 was first detected in humans in Cameroon [16]. In February 2010, the prevalence of pH1N1 was greater than that of seasonal influenza B in humans in Cameroon; however, by May 2010, influenza B had the higher prevalence [17]. Nevertheless, pH1N1 continued to persist in humans in Cameroon at low prevalence as of December 2013 [18]. Cameroon has lower government expenditure on health than neighboring nations and a high prevalence of tuberculosis [19], which is a significant risk factor for respiratory failure during influenza infection [20]. Therefore an epidemic of influenza A or a novel reassortant influenza virus could lead to higher morbidity and mortality than an outbreak in an upper-middle or upper-income economy [21]. In addition, Cameroon is an important stopover and wintering site for migratory birds which may harbor various strains of influenza A virus [22]. Consequently, examining the spillover of influenza A among humans, livestock, and wildlife in Cameroon could provide valuable information on the transmission of the virus with significant implications elsewhere.

Surveillance of pH1N1 in swine to date has focused on genotyping the virus in order to characterize in vivo pathogenicity, and estimate seroprevalence [10,23,24]. However, another important question to be addressed by surveillance efforts is the extent to which agricultural and ecological factors contribute to the emergence and persistence of the virus in domestic animals [25]. Currently, the effects of animal husbandry practices on interspecies transmission are poorly understood. Models suggest that swine raised on large-scale commercial farms, where animals are confined and raised at high densities, could serve as an important source of novel influenza that might trigger a new pandemic by infecting agricultural workers who would serve as a bridge to spread the virus to the rest of the population [26]. Small-scale farms, where swine are largely free-ranging, are common in many developing countries [27]. Yet the role of these small swine farms in facilitating influenza transmission between humans and swine is largely unknown.

We recently detected the first known cases of pH1N1 infected swine in Cameroon as a result of an ongoing collaborative effort to understand the ecology and spillover dynamics of influenza A [10]. A rationale behind these efforts was to identify agricultural production systems and animal husbandry practices that facilitate interspecies transmission of influenza A and to improve biosecurity policies, with a focus on small-scale, low investment and subsistence farms. We build on these findings by assessing how contact rates among species might have influenced the transmission from humans to swine.

## Methods

**Figure 1:**
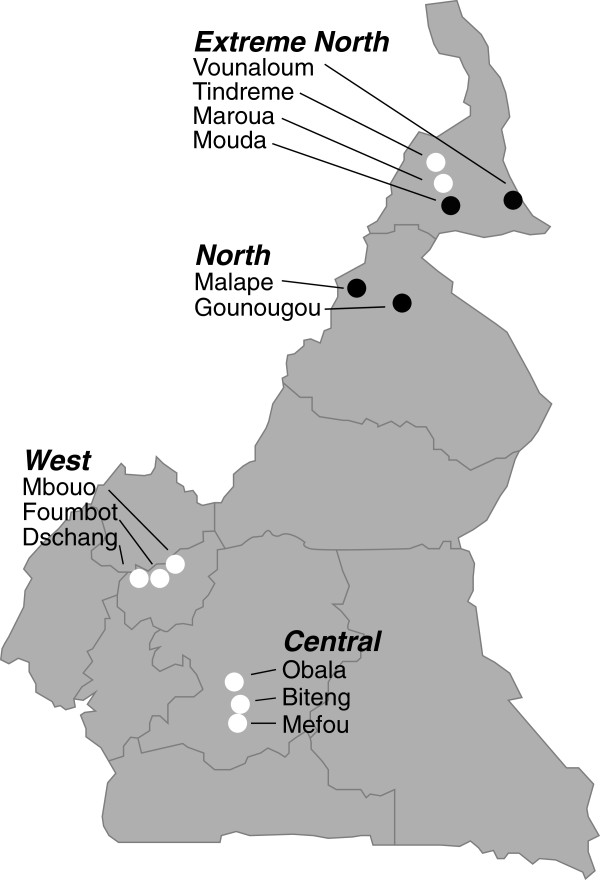
**Presence of pH1N1 at twelve sites in Cameroon.** Sites shown by a white point had no positives for any influenza, those shown by a grey or black point had at least 1 positive for pH1N1. Sites indicated by grey points had positive sera, while those indicated by black points had swine shedding the virus at the time of our sampling.

Between December 2009 and August 2012, we conducted research at 12 sites in Cameroon to assess prevalence rates and risk factors for spillover (Figure 1). Three sites were located in the Central Region, three in West Region, two in North Region and four in the Extreme North Region (regions in Cameroon are administrative units similar to provinces). The sites in northern Cameroon had originally been selected targeting previous influenza A (H5N1) outbreak areas wherever possible [28]. In addition, the North and Extreme North Regions of the country are major wintering areas for migratory waterfowl, the group possibly responsible for the spread of H5N1 from Asia to Europe in 2005 [29,30]. The Central Region comprises the second most populated region in the country, thus we also viewed it as critical to our sampling regime. Sites were selected around the densely populated capital city of Yaoundé because of the expected high levels of interaction between poultry, swine, and humans. However, H5N1 does not appear to have become endemic in the country because our surveillance efforts, since the detection of the initial cases, have not detected H5N1 in wild or domestic birds, and there have been no further reports of this strain of influenza in Cameroon. As our study was initiated, however, pH1N1 was on the rise in humans. We sampled the first six sites in 2010, adding three in 2011 and three in 2012. The sites in 2011 and 2012 were selected to broaden the sampling area and to include regions with both high and low densities of swine. During each visit we sampled domestic poultry, wild birds, and swine to test for active and recent influenza infections. Concurrently, we conducted scan surveys to assess contact rates among humans, domestic animals and wild birds. We screened twice at each site in 2010, but only use the first set of screens in our analysis of risk factors as our second visit to these six sites occurred immediately after a culling event in response to an outbreak of African swine fever and thus scans were not representative of typical conditions.

At each location (village compounds and surrounding farms), we captured and sampled swine, domestic poultry and wild birds for influenza screening. We sampled 325 swine, collecting both nasal swabs and sera whenever possible. Cloacal swabs were collected from 582 domestic poultry (334 chickens, 240 ducks, 4 geese, and 4 turkeys) and from 1479 wild birds captured using passive mist-netting along agricultural edges and natural habitats near villages. We collected sera from 43 ducks, 65 domestic chickens and 91 wild birds. All the swabs were collected and processed following standard protocols [31]. Serological tests were performed according to international standards [31]. Details of methods used to screen for influenza can be found in Njabo et al. [10]. Briefly, paired samples were collected and stored in ethanol and VTM. For some of the passerines, venous blood was also collected in 20-30 μl aliquots using a 50 μl micropipette and sterile tip. The first aliquot of blood was added directly to 450 μl of phosphate buffered saline (PBS). Additional aliquots of blood were sampled and added to the same PBS to achieve a final dilution of 1:10 and mixed briefly by pipetting [32,33]. All the VTM preserved swab samples were tested at the Centre Pasteur in Cameroon for presence of influenza A by real time RT-PCR [28]. The VTM samples were also sent to the OIE/FAO lab in Padova, Italy for confirmation by real time RT-PCR, culturing and virus genome sequencing. All sera samples were screened by competitive ELISA type A assay at the OIE/FAO lab for detection of past exposure to influenza A. The positive swine sera were then tested for the following antibodies by Hemagglutination-inhibition (HI) assays: Eurasian “avian-like” A/Sw/Italy/5766-15/09 (H1N1), triple-reassortant A/Sw/Italy/716/06 (H3N2), and A/Sw/Italy/4660-3/09 (H1N2) and the human influenza viruses pH1N1 (A/California/04/2009) and H1N1 (A/Italy/3983/2009). Sera from birds were also tested by type A ELISA and all the ELISA positive avian sera were tested by HI with avian H5, H7 and H9 antigens. Sampling was conducted under UCLA IACUC protocol 2007-001-13A.

Quantifying contact rates is of considerable interest for assessing risk of transmission and spillover yet standardized methods for quantitative determination of contact rates are lacking. Studies of wild mammals have used radio telemetry to produce a measure of how much home ranges overlap and how often the two animals in question are found utilizing the area of overlap (Utilization Distribution Overlap Index, UDOI) and used this as a proxy for observing direct contact. A recent study in raccoons found UDOI to be a good proxy for direct contact [34]. Assessing the importance of contact rates of wild birds with livestock and poultry in spillover is a major motivating feature of our larger study. As radio telemetry was not feasible for our study we identified a scan sampling technique used for monitoring abundance of wild birds [35,36] as a method that should produce a qualitatively similar measure of overlap as that measured by UDOI and that could be readily adapted for our study. This technique is commonly used to assess abundance of wild birds but has not previously been applied to domestic poultry and swine, nor has it been used, as far as we know, to assess contact rates. The research that forms the basis for this study is part of a collaborative study being conducted in multiple countries with different landscapes and species [37]. We felt this technique could be successfully adapted for the collaborative study because it can be easily scaled to different situations. Scan sampling was conducted in a stratified fashion by placing points in two human dominated land-use categories within each site: 1) within villages, including inside of family compounds, and 2) surrounding agricultural areas on the edge of the urban/village matrix. Within these land-use types at each site we randomly selected 5-10 points at which to conduct scan sampling. Scans consisted of 50 m diameter plots situated such that adjacent perimeters were at least 100 m apart. Fifty meters is a common size plot for this type of scan [36] and readily fit within the scale of the agricultural or village locations while being large enough to minimize altering the natural behavior of the animals in the scans. To make our sampling more representative, each plot was surveyed 5-10 times between 06:00 and 18:00, and scans at any one point were conducted at least one hour apart. To conduct each survey, two observers entered a plot and silently waited at the center before beginning the count. After one minute, one observer would begin scanning while the other recorded. Scans were started by first facing north, and proceeding in a clockwise direction, coming full circle over a period of 5-10 minutes depending on environmental complexity and the number of animals in the scan. The locations and behaviors of all humans and wild or domestic animals in the plot were recorded, and for domestic animals, whether they were free-ranging, penned or caged. We also recorded any human and animal activity that could be observed in the immediate vicinity of the plots. Observers were trained to visually estimate the approximate location of objects in the plots with <10% error prior to beginning the surveys. Surveys were conducted in compliance with the Helsinki Declaration under protocol #11-000934, approved by the UCLA Institutional Review Board.

As our surveys resulted in 29 predictor variables, many of which were highly correlated (Additional file 1: Figure S1), we analyzed the data using random forest classification models as implemented in the package randomForest in R [38,39]. We ran 5000 trees within each random forest run, removing the least important variables after each run until we identified the “best” model (“best” was defined as the model with the highest OOB, or out of bag, variance explained). Unlike standard statistical models, this modeling approach makes no a priori assumptions about the relationship between the response and predictor variables, can make accurate predictions from ‘wide’ datasets containing more predictors than observations, and are less susceptible to spatial autocorrelation [38,40,41]. The basis of the model is a decision tree, which uses a binary recursive partitioning procedure to measure the amount of variation in a response variable explained by each predictor variable in the model, splitting the response variable successively by the variable explaining the majority of the remaining variance. Random forests use a randomized bootstrapping method in which each tree is constructed using a randomly selected subset of both the observations and predictor variables. This bootstrapping provides an intrinsic cross-validation which reduces the likelihood of over-fitting relative to more standard models [38,42]. The error rate of the model is based on the combined error rate of all the bootstrap iterations when the data not included in that iteration is predicted using the tree from the current iteration. Variables are ranked in order of importance as determined by the reduction in predictive accuracy of the model when that variable is permuted randomly. We defined contact as the pairwise co-occurrence of two individuals of a species pair within the same 50 m point during the same scan. Thus contact rates were calculated by multiplying the number of each species in each scan by the number of each other species in the scan. Mean contact rates were then calculated for each species pair for each site. Our predictor variables were the set of pairwise contact rates that occurred between humans and swine (free or penned), swine and avian species (both wild and domestic), and humans and avian species. Our response variable was a binary variable that reflected whether influenza was detected or undetected at a site. Because the number of swine we were able to test for influenza varied among sites we also included sampling effort among our predictor variables. As humans were rarely recorded within our plots, and may have avoided them, we included both humans within the plots and those observed in the immediate vicinity in the analyses.

## Results

### Sampling and screening

Swine tested positive for pH1N1 at four of our 12 sites (Figure 1). Active cases in which the virus was still present were detected using real time RT-PCR on the VTM nasal swabs (GenBank accession numbers JF707781-JF707788) and were extremely rare; only one swine at each of two sites tested positive for active infections and only during early 2010. All swine testing positive in the ELISA assays were tested by HI with swine flu antigens H1N1, H1N2, H3N2, and pH1N1 and had HI titers consistent with pH1N1 (median = 2560, range 160-20480, negative titer = 40). One of these swine also had a titer of 640 (two fold the negative titer of 80) for H3N2, but this same swine had a pH1N1 titer of 2560 (6 fold the negative titer of 40 for this strain).

Sera from domestic birds all tested negative by type A ELISA with five exceptions from chickens (four at Tindreme and one at Mouda, both in the Extreme North Region). Four of these sera provided doubtful results in ELISA. All the ELISA positive yet doubtful avian sera were tested by HI with avian H5, H7 and H9 antigens. All gave negative results.

### Species abundance and contact rates

**Figure 2:**
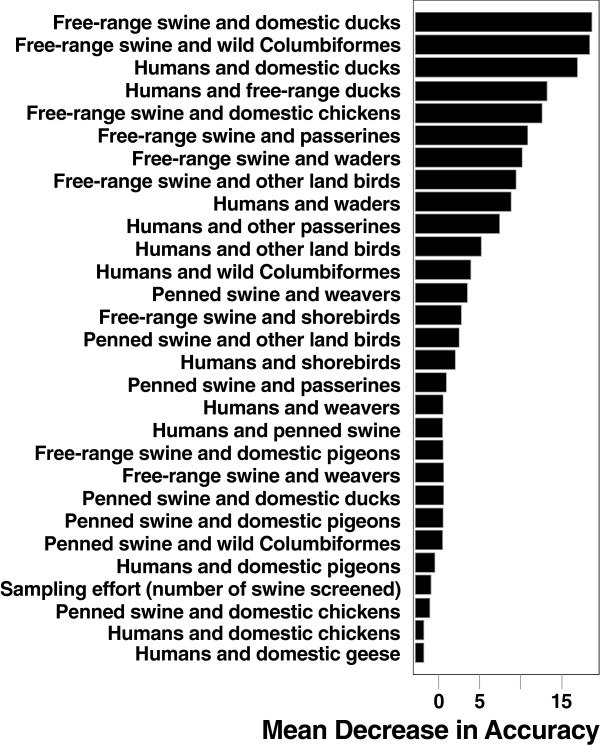
**Variable importance from random forest analyses.** All variables included in the model are shown listed from top to bottom in order of importance. Variables are the pairwise contact rate (as defined in the text) between the two taxa listed. A variable’s importance is determined by the decrease in the predictive accuracy of the model when that variable is permuted. Waders include all water-associated birds excluding Anseriformes and Charadriformes (shorebirds). Other land birds include all terrestrial birds excluding Passeriformes, Columbiformes and Galliformes and Village Weavers.

A salient difference among sites was whether swine were kept penned or allowed to range freely. Swine were free ranging at 5 of the 6 northern sites, but were nearly always penned in the other sites (Table 1, Figure 1). Domestic poultry were allowed to range freely at all sampled sites. Tree classification using the full set of variables resulted in a single split wherein spillover is predicted to occur at sites with a human-duck contact rate of 1.3027 or greater. Random forest classification analyses indicated that the top three predictors of pH1N1 being present in swine were contact rates between ducks and free-ranging swine, contact rates between swine and wild columbiform birds, and contact rates between humans and ducks (Figure 2). Seven of the top 10 predictors involved free-ranging swine (Figure 2, Table 1). The error rate of the full classification model with all variables was 8.33%. In the full model, sites in which we did not detect influenza were always correctly classified, but one of the four sites in which we did detect influenza was consistently misclassified as a non-influenza site. A model using only the top three predictors, however, successfully predicted both positive and negative sites 100% of the time, suggesting that some variables served only to confound classifications when used as predictors. Table 2 shows the effect of allowing swine to range freely on contact with ducks and Columbiformes. In most sites where swine were penned, swine and these two avian groups did not co-occur within the same scan, or even within the same plot at different times. Where swine were free-ranging they often co-occurred in the same plot either during the same scan or in the same plot at another time. Additionally, behavioral notes taken during the scans by observers included many instances of close contact between swine and other species, all but one of which occurred in sites with free-ranging swine. These included three swine eating a dead duck (Malape), 11 cattle egret following swine in a marsh area (Gounougou), swine foraging in a hut (Vounaloum), and swine sleeping in a hut with humans (Malape). The only such observation including penned swine was of 7 chickens feeding under an elevated pen with swine in it (Obala).

**Table 1:** Values of the three most important random forest variables by site

**Region**	**Location**	**Number of scans conducted at site**	**Mean contact rate between free swine and ducks**	**Mean contact rate between free swine and Columbiformes**	**Mean contact rate between humans and ducks**
Extreme North	**Vounaloum**	**60**	**4.06**	**0.70**	**20.55**
	Tindreme	92	0.00	0.00	0.00
	Maroua	160	0.00	0.00	2.20
	**Mouda**	**158**	**0.14**	**0.08**	**13.10**
North	**Malape**	**75**	**0.08**	**0.29**	**10.08**
	**Gounougou**	**150**	**0.49**	**0.17**	**3.36**
West	Mbouo	160	0.00	0.00	0.26
	Foumbot	159	0.00	0.00	0.00
	Dschang	159	0.00	0.00	0.18
Central	Obala	75	0.00	0.00	0.08
	Biteng	119	0.00	0.00	0.00
	Mefou	74	0.00	0.00	0.41

Bold: sites where influenza was detected.

**Table 2:** Minimum proximities observed between swine and a) ducks and b) columbiformes at each site

**Region**	**Location**	**Swine free-ranging or penned**	**Minimum proximity of swine to domestic ducks (m)**	**Minimum proximity of swine to columbiform birds (m)**
Extreme North	**Vounaloum**	**Free-ranging**	**0.4**	**1.3**
	Tindreme	Penned	.	9.6
	Maroua	Penned	>25	>25
	**Mouda**	**Free-ranging**	**4.7**	**4.3**
North	**Malape**	**Free-ranging**	**0.0**	**5.9**
	**Gounougou**	**Free-ranging**	**1.9**	**0.2**
West	Mbouo	Penned	>25	>25
	Foumbot	Penned	.	>25
	Dschang	Penned	>25	.
Central	Obala	Usually penned	0.8	>25
	Biteng	Penned	>25	>25
	Mefou	Usually penned	>25	24.7

Bold: sites at which influenza was detected. At the sites for which proximity is missing, we observed no Columbiformes or ducks in our scans or elsewhere during our visit to the site.

## Discussion

Our results show that animal husbandry practices such as the manner in which animals are housed may influence contact rates, and as a consequence, spillover of influenza. Other studies have found that whether or not animals are kept separate can influence transmission of influenza among species [43,44], but these findings are typically based on qualitative observations and self-reporting by locals. Our study is unique in providing a quantitative assessment of the relationship between contact rates and spillover of influenza. Our results suggest that influenza is more likely to spillover from species to species when farm animals are allowed to roam freely. The majority of the important variables for predicting influenza in swine involved contact between free-roaming swine and other species, and the data are highly suggestive that spillover to swine is more likely where swine are free-ranging. This is because free-ranging swine are likely to come into closer contact with other species than penned swine, either directly or through contact with feces or even dead animals (Table 2). As influenza A can replicate in the intestines of both birds and mammals (including humans), one hypothesis is that swine acquired pH1N1 by consuming human feces containing the virus, though this awaits confirmation [45-48]. Contact with feces may have contributed to spillover in the Extreme North Region as overall hygiene is poor and the people in this region regularly practice open defecation rather than using latrines. Free-ranging swine were observed eating human feces in this region. Research shows that lack of education and resources as well as cultural preferences all impede the construction and use of sanitary latrines [49,50]. The results of this study underscore that to reduce disease outbreaks in this region sanitation and hygiene intervention must be implemented.

The exact nature of the interspecific transmission routes is more difficult to discern from our data, for two reasons. First, contact rates between humans and other species were not easily quantified. Phylogenetic analysis indicates high similarity between pH1N1 isolated from Cameroonian swine and humans, and anecdotal evidence suggests direct human to swine transmission may be possible. However, as noted in the methods, humans may have avoided entering the area of our scans as they were recorded in scans at only two sites, and then only rarely. Thus to include humans in the study at all required that we include those humans observed outside the scans. Second, our data point to domestic ducks and wild Columbiformes as possible intermediaries. However the correlations among the top three most important contact rates revealed by random forest analyses are 0.91 (duck-swine and swine-columbiformes), 0.78 (human-duck and swine-duck) and 0.88 (swine-columbiformes and human-duck), making it difficult to determine which variable is likely to be causal and which are merely correlative. We did not detect any cases of influenza in domestic ducks or Columbiformes, nor did we encounter any ducks or Columbiformes that showed signs of disease. However, in some cases, domestic and wild ducks may support influenza A infections without showing symptoms [51]. Our screening of birds primarily involved real time RT-PCR of cloacal swabs, which will only detect influenza during the short period of time when the virus is being shed. Only 43 ducks were screened by ELISA and we had no sera from Columbiformes.

The regional differences we observed may suggest that swine in the North and Extreme North were infected simply because human cases of pH1N1 occurred in this region and not elsewhere. However, this is unlikely given the first human case in Cameroon was reported from the Central Region, and the first apparent case of human-human transmission occurred in the Central Region (R. Njouom, personal communication). It has long been appreciated that geographic differences in influenza prevalence among swine may arise in response to ecological factors [52]. For example, differences in weather conditions between the North and Central Region might partially explain the differences in pH1N1 prevalence observed. During our sampling period, the Harmattan trade wind blows across northern Cameroon, resulting in a large decrease in humidity [53]. Low humidity is one of the main environmental drivers of influenza transmissibility and prevalence in laboratory experiments [54]. Dry winds could create conditions favorable to the persistence of the virus in dryer areas of northern Cameroon, leading to increased transmission among swine, and the high prevalence that we detected. Such weather differences might also help explain the complete lack of pH1N1 we found in the humid Central and West Regions, which the Harmattan does not reach.

## Conclusions

We conclude that free-roaming swine are a significant risk factor for the spillover and spread of influenza in Cameroon. The practice of allowing swine to roam free, often combined with poor hygienic practices, may be an important factor in spillover of pH1N1 from humans to swine in northern Cameroon.

## Competing interests

The authors declare they have no competing interests.

## Authors’ contributions

BL, TBS, KYN and AC designed the field methods. KYN and AC oversaw field sampling, sample analysis, and surveys. BL oversaw data entry, conducted the analyses presented in the paper and drafted the manuscript. TF provided GISAID data. KYN, AC, RJH, TF and TBS helped draft parts of the manuscript. All authors read and approved the final manuscript.

## Supplementary Material

Additional file 1 Correlations Among Predictor Variables. Click here for file
