# Supplementary figures and images for: Spillover of pH1N1 to swine in Cameroon: an investigation of risk factors
Source: BMC Vet Res. 2014 Mar 4;10:55. doi: 10.1186/1746-6148-10-55 (PMC4016523; doi:10.1186/1746-6148-10-55)

Correlations Among Predictor Variables

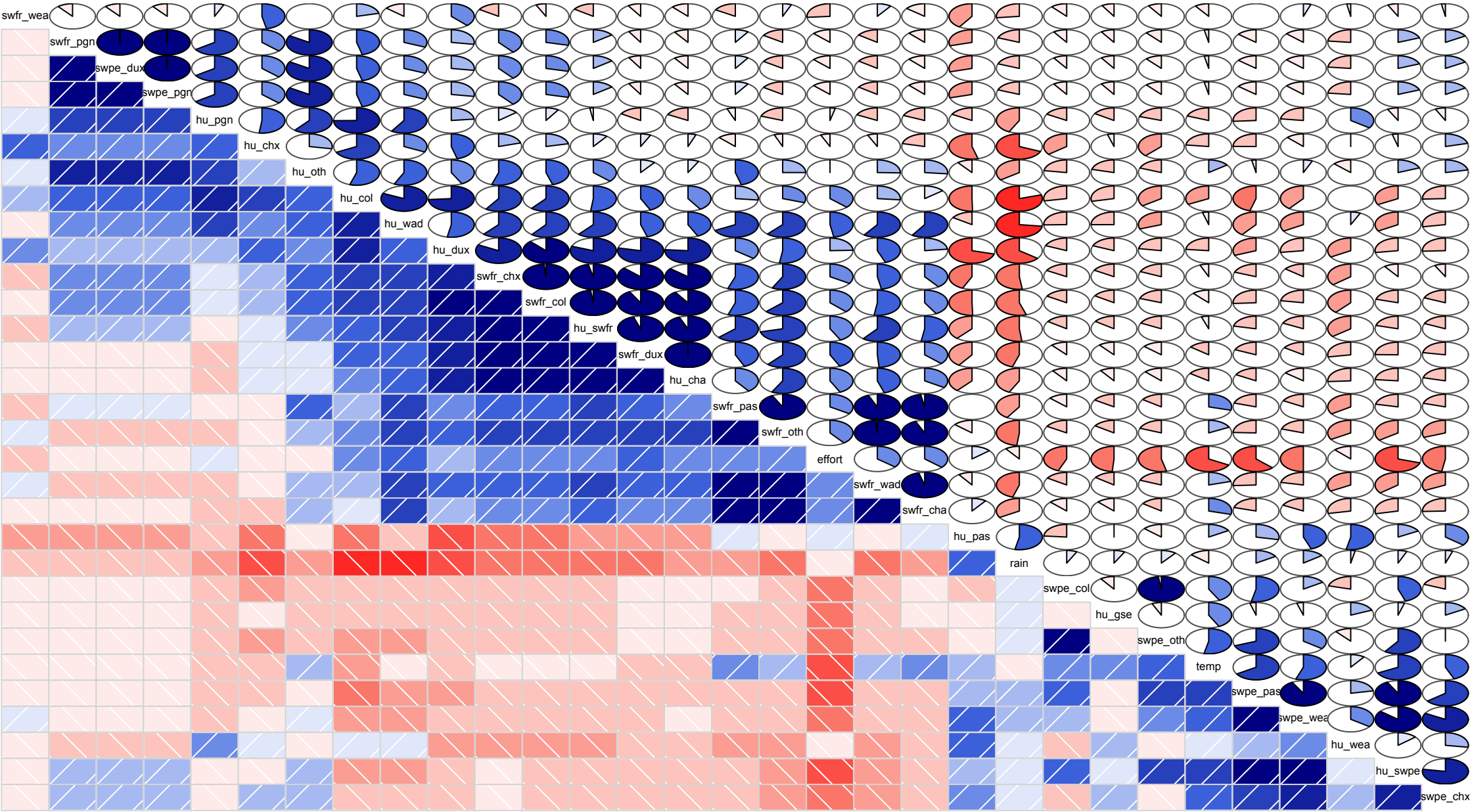

Supplement: Additional file 1 — Correlations Among Predictor Variables. [file 1746-6148-10-55-S1.pdf]
